# The Antimicrobial Activity of Micron-Thin Sol–Gel Films Loaded with Linezolid and Cefoxitin for Local Prevention of Orthopedic Prosthesis-Related Infections

**DOI:** 10.3390/gels9030176

**Published:** 2023-02-23

**Authors:** Beatriz Toirac, John Jairo Aguilera-Correa, Aranzazu Mediero, Jaime Esteban, Antonia Jiménez-Morales

**Affiliations:** 1 Materials Science and Engineering and Chemical Engineering Department, Carlos III University of Madrid, 28911 Madrid, Spain; 2 Clinical Microbiology Department, IIS-Fundación Jiménez Díaz, UAM, 28040 Madrid, Spain; 3 CIBERINFEC-Consorcio Centro de Investigación Biomédica en Red (CIBER) de Enfermedades Infecciosas, 28029 Madrid, Spain; 4 Bone and Joint Unit, IIS-Fundación Jiménez Díaz, UAM, 28040 Madrid, Spain; 5 Alvaro Alonso Barba Technological Institute of Chemistry and Materials, Carlos III University of Madrid, 28911 Madrid, Spain

**Keywords:** nanogels, sol–gel coating, linezolid, cefoxitin, Prosthesis-Related Infections, *Staphylococcus epidermidis*, *Staphylococcus aureus*, *Escherichia coli*, Electrochemical Impedance Spectroscopy

## Abstract

Orthopedic prosthesis-related infections (OPRI) are an essential health concern. OPRI prevention is a priority and a preferred option over dealing with poor prognosis and high-cost treatments. Micron-thin sol–gel films have been noted for a continuous and effective local delivery system. This study aimed to perform a comprehensive in vitro evaluation of a novel hybrid organic–inorganic sol–gel coating developed from a mixture of organopolysiloxanes and organophosphite and loaded with different concentrations of linezolid and/or cefoxitin. The kinetics of degradation and antibiotics release from the coatings were measured. The inhibition of biofilm formation of the coatings against *Staphylococcus aureus*, *S. epidermidis,* and *Escherichia coli* strains was studied, as well as the cell viability and proliferation of MC3T3-E1 osteoblasts. The microbiological assays demonstrated that sol–gel coatings inhibited the biofilm formation of the evaluated *Staphylococcus* species; however, no inhibition of the *E. coli* strain was achieved. A synergistic effect of the coating loaded with both antibiotics was observed against *S. aureus*. The cell studies showed that the sol–gels did not compromise cell viability and proliferation. In conclusion, these coatings represent an innovative therapeutic strategy with potential clinical use to prevent staphylococcal OPRI.

## 1. Introduction

The use of orthopedic implants has increased over recent decades [1]; though the incidence of orthopedic prosthesis-related infections (OPRI) is low (1–2%), the number of cases increases proportionally with the number of prostheses, making OPRI a vital health concern [2]. Such infections are characterized by poor patient prognosis associated with comorbidity and high medical cost treatments. The prevention of OPRI is a priority and a preferred option over dealing with complex and expensive treatment.

An area gaining increasing interest in preventing OPRI is the controlled release and local delivery of antibiotics from suitable carriers. Access to the site of infection by antibacterial agents administered orally or intravenously is limited due to bone necrosis and poor vascular perfusion that often occurs in complicated OPRI [3]. The local release provides more efficient delivery of higher concentrations of antibiotics to the site of infection, many times more than the minimum inhibitory concentration (MIC) while minimizing serum concentrations and systemic toxicity associated with traditional methods of intravenous antibiotics. The execution of this approach in conjunction with systemic therapy will produce a result superior to systemic therapy alone, as has already been observed in two-stage arthroplasty revision procedures [4].

However, due to the growing development of antibiotic resistance, efforts have been made to seek alternatives, beginning with antibiotic combination therapy, addressed in this work [5]. Currently, numerous strategies aim to prevent prosthesis-related infections. With the use of antibiotics being the most widespread strategy, other alternatives are gaining interest, such as implant surface modification [6], cationic biocidal polymers, antifouling coatings [7], coatings based on metal-oxide nanoparticles, antimicrobial peptides (AMPs) [5,7], and bacteriophage therapy [5,8], among others [9,10,11]. Specifically, in the area of release systems, major progress has been made in chlorhexidine-releasing, silver-releasing, furanone-releasing, and nitric oxide-releasing coatings [7]. Although many of these alternatives are promising and could have high relevance in the coming years, they are currently in the early stages of the investigation, and many of them have not reached clinical trials, not even animal models in some cases [7,12].

Micron-thin sol–gel films have been noted as a continuous and effective local delivery system for orthopedic instruments to prevent and treat OPRI [13]. These types of coatings can not only prevent OPRI but could also be used in other devices where this type of infection occurs. For staphylococcal infections, the application field is very wide, including not only infections related to orthopedic implants but also breast implants, heart valves, cardiac pacemakers, endotracheal tubes, urinary catheters, central venous catheters, and prosthetic devices for erectile dysfunction [7,14].

The preparation of coatings by sol–gel technology offers multiple advantages since it is one of the most simple and versatile synthesis methods, allowing the control of the final structure of the coating. Its degradation by-products are not toxic to humans. The possibility of low-temperature processing allows the incorporation of thermolabile molecules without their denaturation. These biomolecules are encapsulated within the sol–gel network and their release is linked to the coating degradation, thus achieving a sustained and controlled biomolecule release [15,16,17]. However, it should be considered that the concentration of the added biomolecule is limited by the final physical properties sought in the coating. Using antibiotics as a biofunctionalizer of these systems ensures the action mechanism to combat the bacteria but increases the possibility of the emergence of multidrug-resistant microorganisms [14]. This option is minimized as it is a local release where the antibiotic will be placed directly at the implant/periprosthetic tissue interface [8]. Another precaution taken to avoid this possibility is combination therapy [8,18,19]. The long-term effectiveness of the drugs incorporated in these systems has yet to be demonstrated [7].

The first use of sol–gel technology as an implant coating for use in antibiotic delivery dates back to 2007 when S. Radin introduced vancomycin in the sol–gel network [20] and demonstrated its effectiveness in combating osteomyelitis [21]. The study of a sol–gel film loaded with both an antibiotic and an adjuvant, namely vancomycin and farnesol, to treat methicillin-resistant *Staphylococcus aureus* (MRSA) infections has also been reported [22]. A sol–gel coating releasing triclosan was studied to prevent pin tract and periprosthetic infections [23,24]. Nichol et al. reported a sol–gel coating loaded with gentamicin for bioceramic-coated cementless arthroplasty material [25]. In previous works, we have developed two sol–gel coatings with the incorporation of fluconazole and anidulafungin [26]. Its antifungal effectiveness was demonstrated in in vitro [27] and in vivo [28] studies in subsequent reports. In addition, in vitro and in vivo studies were performed on a moxifloxacin-loaded sol–gel coating for the prevention of bacterial prosthetic joint infection [29] and bacterial intravenous catheter-related infection [30]. Despite this, there is no evidence of reports based on sol–gel films loaded with two antibiotics simultaneously and their impact on antibacterial effectiveness, as proposed in this study.

The combination of two antibiotics instead of monotherapy is widely used when a single antibiotic is not expected to have a spectrum broad enough to cover all potential pathogens [31]. Furthermore, combination therapy offers advantages such as the prevention of resistance emergence [18,19], the synergistic action of the combination against a specific pathogen [32], or the medication of a polymicrobial infection not treatable with a single drug [33,34]. In the treatment of staphylococcal infections, the most common strains in OPRI [1], rifampin is preferred as the standard combination partner [35], but its activity against other organisms remains unclear [36].

The present study is focused on the microbiological evaluation, as well as the study of the coating degradation and antibiotics release rate, of coatings biofunctionalized with different concentrations of linezolid (LNZ) and cefoxitin (FOX) to locally prevent prosthesis-related infections.

While broadening the antibacterial spectrum of the coatings is the main goal of combining these two antibiotics, synergistic action is expected in the treatment of gram-positive bacterial infections, since the mechanism of action of both antibiotics is different. Linezolid is the first antibiotic of the oxazolidinone group and significantly surpasses the MIC_90_ of enterococci and staphylococci [36] in current resistance patterns. By decreasing the susceptibility of vancomycin to methicillin-resistant staphylococci, linezolid can potentially be used in bone and joint infections [36,37]. However, recent studies of trends in OPRI etiology report a statistically significant increase in the proportion of infections caused by Gram-negative bacilli in recent years [1]. The antibacterial spectrum of cefoxitin includes the aerobic Gram-positive cocci organisms commonly encountered in OPRI, many aerobic Gram-negative bacilli, and anaerobic strains, which allows cefoxitin to provide adequate antibacterial coverage. For these reasons, cefoxitin has been widely and successfully used for the prevention and treatment of a variety of difficult-to-treat postoperative infections in orthopedic surgery, including some polymicrobial etiology [38,39,40,41].

The sol–gel coating loaded with linezolid and cefoxitin will be able to release these antibiotics and fight infections of Gram-positive and Gram-negative strains.

## 2. Results and Discussion

### 2.1. Synthesis and Samples Preparation

**Figure 1:**
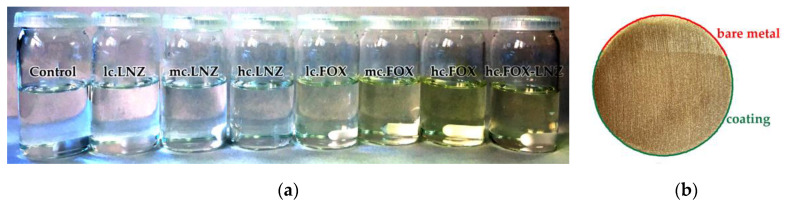
(**a**) Production batch of the sol–gels of all the formulations analyzed (antibiotic-free coating: Control; LNZ-loaded coatings: lc.LNZ, mc.LNZ, and hc.LNZ; FOX-loaded coatings: lc.FOX, mc.FOX, and hc.FOX; and coating loaded with both antibiotics: hc.FOX-LNZ). (**b**) Example of a coating deposited on a titanium disc produced by powder metallurgical route (part of the metal area was left uncoated for exemplification and visual support).

After 24 h of the reaction, the sols obtained in all formulations were translucent and without phase separation, as shown in Figure 1a. The adequate viscosity of the sols facilitated the correct application on the substrate. The dried coatings on the powder metallurgical titanium were simple-sight inspected, without imperfections such as cracks or pores (Figure 1b). A complete characterization of the synthesis and the surface of these coatings can be read in a previous report [42].

### 2.2. Coating Degradation Study

**Figure 2:**
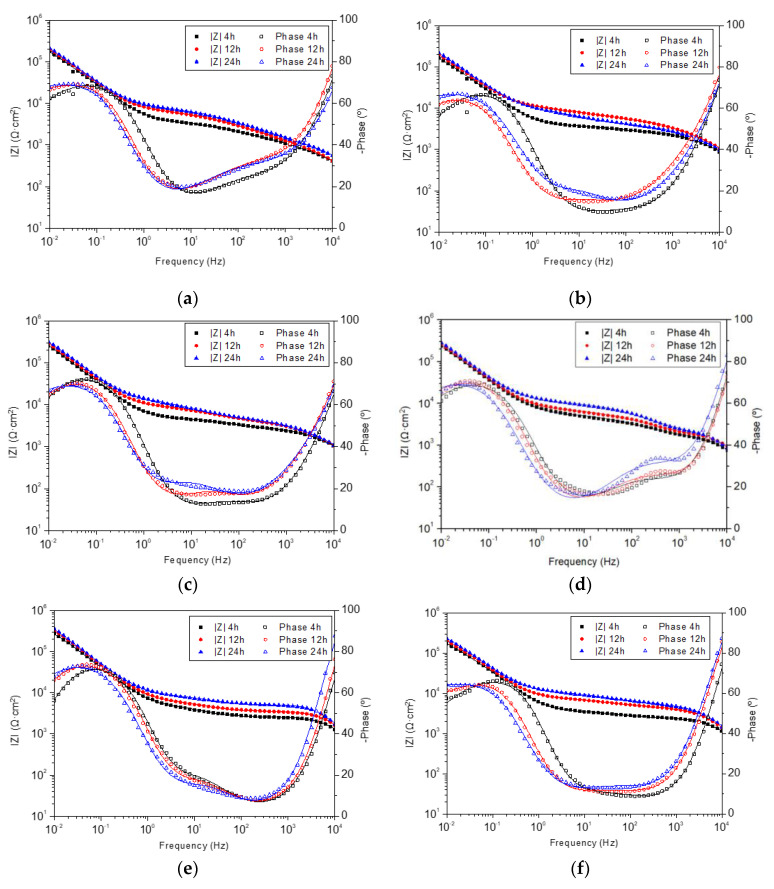

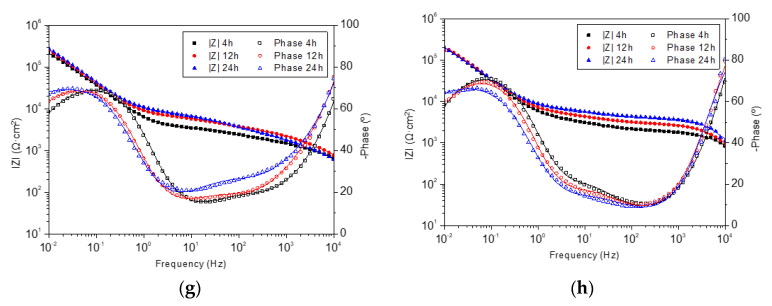
Evolution of Bode spectra with the exposition time on EIS tests. (**a**) Control, (**b**) lc.FOX, (**c**) mc.FOX, (**d**) hc.FOX, (**e**) lc.LNZ, (**f**) mc.LNZ, (**g**) hc.LNZ, and (**h**) hc.FOX-LNZ.

Figure 2 depicts the Bode plots from Electrochemical Impedance Spectroscopy (EIS) test results, at 4, 12, and 24 h of sample immersion in PBS at 37 °C. In all the systems, the absence of great variations was evidenced during the 24 h period, labeling them as stable systems. Despite this, a slightly increasing trend of the average impedance from 4 h to 24 h was revealed, indicating an enhancement of corrosion barrier properties with evolution over time. In all formulations, the same order of magnitude in the impedance module value at 10^−2^ Hz was obtained in the module Bode plots, with very similar values (≈2 × 10^5^ Ω∙cm^2^).

In the phase angle Bode plots, the phase peak located at low-mid frequencies behaved similarly in all systems. A leftward shift of this peak was observed (except in lc.FOX), which represents a less electrochemically active area [43].

The impedance module value at frequencies related to coatings (≈100 Hz) [26] evidenced an increase with the immersion time, except in the lc.FOX sample, which could be the result of water uptake or due to their capacitance increase, meaning an increase in the active area, possibly due to the degradation of the coatings, eroding its surface and exposing a greater area to the electrolyte [44].

**Figure 3:**
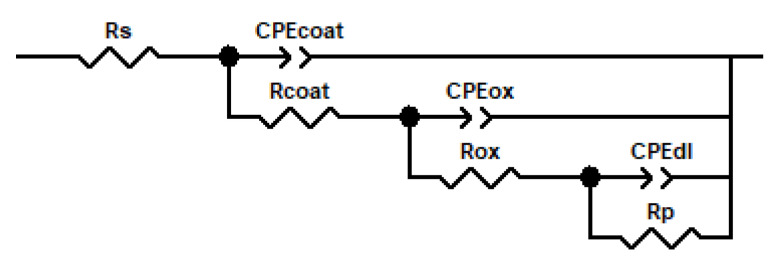
Equivalent electrical circuit and its passive elements used to fit the curves obtained from the electrochemical impedance tests.

The interpretation of the curves obtained in the EIS tests was performed by fitting them to equivalent electrical circuits. The electrical elements of the chosen circuit depict the dielectric properties of the system layers and the electrochemical processes occurring in the system. Figure 3 shows the equivalent electrical model used, and the electrical elements that constitute it are listed below. This model of three time constants is widely used in porous coatings that allow the electrolyte to reach the coating/metal substrate interface, and as a consequence of this interaction, a passive layer is formed [45]. R_coat_ (Coating Resistance) and CPE_coat_ (Coating Capacitance Constant Phase Element) refer to coating-related processes occurring at high frequencies. In this manner, R_coat_ is associated with the porosity and deterioration of the coating and CPE_coat_ refers to the water uptake into the coating. R_ox_ (Oxide Layer Resistance) and CPE_ox_ (Oxide Layer Capacitance Constant Phase Element) are related to the oxide layer that can appear on the metal surface. They can be determined at intermediate frequencies and provide information on the formation and growth trend of the oxide layer, as well as on its stability. Lastly, R_p_ (Polarization Resistance) and CPE_dl_ (Double-Layer Capacitance Constant Phase Element) are processes localized at low frequencies, referred to as changes in the coating/substrate interface. While R_p_ is associated with the polarization resistance of the interface between the outer layers and the metallic substrate, CPE_dl_ refers to the uncoupling of the coating and the initiation of the corrosion process at the interface [45].

**Figure 4:**
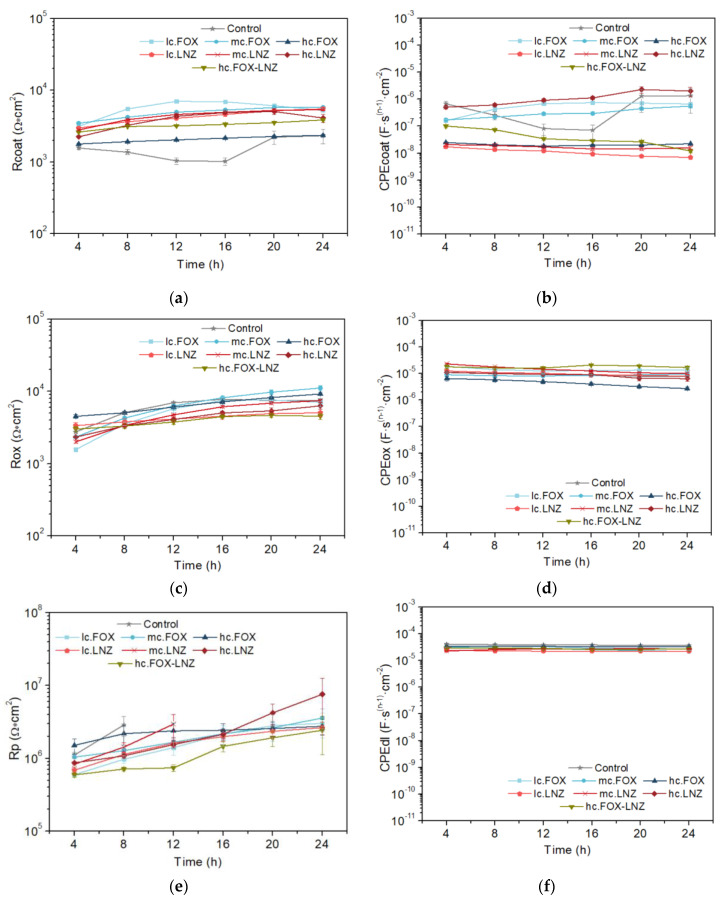
Evolution vs. time of exposure in the electrolyte of (**a**) coating resistance, R_coat_; (**b**) constant phase element of the coating, CPE_coat_ (0.53 < *n* < 1); (**c**) resistance of oxide layer (corrosion products), R_ox_; (**d**) constant phase element of corrosion layer, CPE_ox_ (0.55 < *n* < 0.83); (**e**) polarization resistance, R_p_; and (**f**) double layer constant phase element, CPE_dl_ (0.86 < *n* < 1).

The chi-squared parameter (χ^2^) obtained in the fits of all the systems was usually less than 0.003. The *n* values in the phase constant elements (CPE) used in the modeling are an indicator of the closeness of this element to the behavior of a capacitor, with the value of 1 being considered the response of an ideal capacitor. In the adjustment, anomalous values of the pair R_p_/CPE_dl_ of the last time constant were obtained. Figure 4 shows the evolution of the passive elements of the equivalent circuit with the immersion time in the electrolyte for each studied system. While R_p_ and CPE_dl_ plots are represented, some points were omitted due to these inconsistencies.

The R_coat_ parameter (coating resistance) displayed an increase with immersion time in almost all systems and timepoints. Exceptions were observed, some systems remained almost constant throughout the test (hc.FOX-LNZ) or decreased from certain timepoints (lc.FOX, mc.FOX, and hc.LNZ). The decrease in R_coat_ is proportional to the coating permeability and its increase is related to the blockage of the coating pores according to the substrate passivation [26]. The coating resistances of all the formulations are in the order of 10^4^ Ω∙cm^2^.

The water content that can load the coating is directly connected to its capacitance values, CPE_coat_. Stability was common in all systems, although their CPE_coat_ values varied by orders of magnitude. These values are associated with water uptake, which in turn are related to the number and extension of pores or capillary channels perpendicular to the substrate surface. Although thickness differences between coatings were kept to a minimum (available in [42]), this factor could also influence the CPE_coat_ values. Noteworthily, those specimens whose R_coat_ decreased at a certain timepoint mostly showed increasing behavior in the CPE_coat_. A higher porosity of the coating led to a decrease in its resistance.

The analysis of the difference in orders of magnitude of the capacitance values between the coatings is necessary. The porosity in the coatings loaded with LNZ seemed to be proportional to the antibiotic concentration, while in FOX coatings, it had an inverse behavior. This observation indicates that cefoxitin somehow favors the crosslinking of the sol–gel network since the expected result would be the behavior obtained in LNZ coatings. Supporting this reasoning, the coating loaded with both antibiotics has an intermediate capacitance value.

The oxide layer resistance (R_ox_) was augmented in all systems, with values close to each other (10^3^–10^4^ Ω∙cm^2^). This behavior is due to titanium passivation. The documented differences between systems could be influenced by the permeability of each coating and, therefore, the total surface area of the titanium in contact with the electrolyte.

In general, CPE_ox_ remained stable with very close values in all systems, with a slight decrease indicating the formation of an increasingly less permeable oxide layer.

The polarization resistance (R_p_) illustrated an increasing trend in all systems. This is supported by the titanium passivation and the plugging of the coating pores by the oxide layer formation. The double-layer capacitance area (CPE_dl_) remained stable throughout the test in all systems. No differences were found between the systems. 

**Figure 5:**
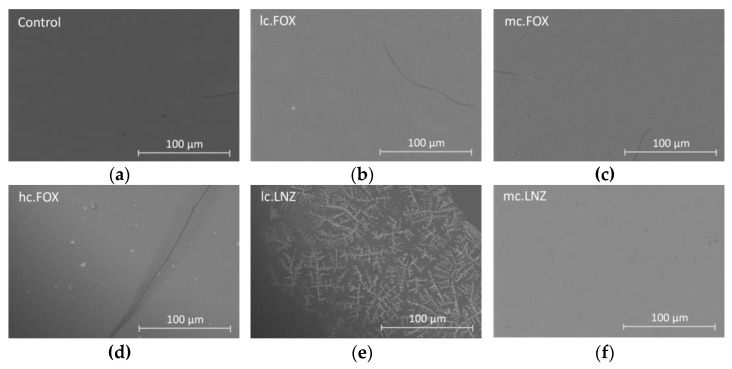

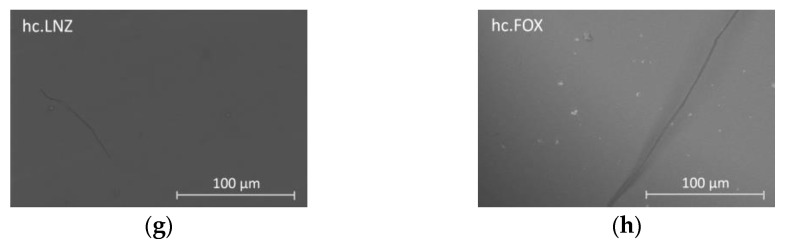
Micrographs obtained with CBS detector by SEM of the (**a**) Control, (**b**) lc.FOX, (**c**) mc.FOX, (**d**) hc.FOX, (**e**) lc.LNZ, (**f**) mc.LNZ, (**g**) hc.LNZ, and (**h**) hc.FOX-LNZ coatings after 24 h of immersion in PBS at 37 °C.

Inspection of the surface of the coatings after completion of the EIS tests complemented the analysis of the test results. SEM images were taken for each sample (Figure 5).

The images are consistent with the results obtained in the EIS tests. After 24 h of immersion in the electrolyte, the R_coat_ values continued to be very close to those values at the start of exposure. However, all the samples show isolated cracks as a sign of deterioration. Larger cracks are observed in hc.FOX and hc.FOX-LNZ, with these coatings and the Control having the lowest R_coat_ values.

### 2.3. Antibiotics Release Study

**Figure 6:**
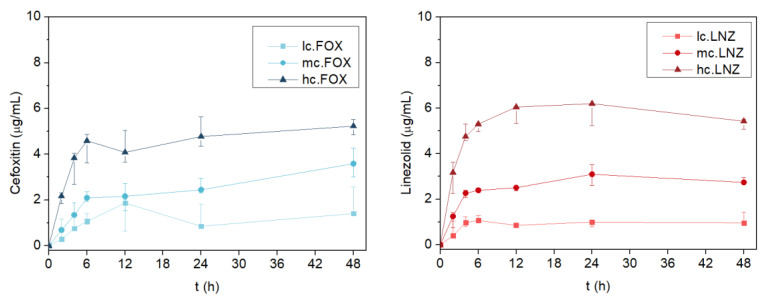
Kinetics of (**left**) cefoxitin and (**right**) linezolid released over time determined by UV-VIS absorbance. Data are represented as the median and interquartile range of the amount of antibiotic measured in three replicates.

The release of both antibiotics followed a similar pattern (Figure 6). A concentration-dependent release of each formulation was evidenced in both antibiotics. Most of the drug was released within the first 6 h (R^2^ = 0.9550 for cefoxitin and R^2^ = 0.8285 for linezolid) following linear behavior. The release kinetics in the coatings loaded with linezolid revealed a decreasing trend in the last hours of measurement. The degradation of this antibiotic is accelerated by increasing the temperature and exposure time in the solution, and this implies a coloration change [46,47]. The color change explains the decrease in the concentration read through absorbance. 

Table 1 lists the constant release rate reached up to 6 h for each coating, as well as their maximum concentrations released. From 6 to 48 h, the release of both antibiotics stabilized and stayed constant over time (*p* = 0.599 for lc.FOX, *p* = 0.053 for mc.FOX, *p* = 0.313 for hc.FOX, *p* = 0.168 for lc.LNZ, and *p* = 0.306 for hc.LNZ; Kruskal–Wallis test) excluding mc.LNZ, which stayed constant from 12 to 48 h (*p* = 0.061 for the Kruskal–Wallis test).

Only two other studies have been found in the literature that address the cefoxitin release in coatings for biomedical applications. The results obtained in both were contradictory. The first one dates to 2004, when Kim and co-workers incorporated this antibiotic into poly(lactide-co-glycolide) (PLGA) scaffolds. The drug was encapsulated by adding the amphiphilic poly(ethylene glycol)-b-poly(lactide) (PEG-b-PLA) diblock copolymer to the solution. The release profile was evaluated by UV-VIS spectroscopy. As a result, approximately 70% of all the loaded antibiotic was released within the first hour, and the rest was released over one week [48]. Despite demonstrating antibacterial effectiveness, burst release is an undesirable effect.

The second was reported in 2016 by Back et al., where they incorporated various antibiotics into poly(D,L-lactide) (PDLLA) coatings. The release determination was confirmed by liquid chromatography with mass spectrometry (LC-MS/MS). The first burst release of cefoxitin is reported, and immediately after this, there is a considerable decrease in the amount of antibiotic detected due to antibiotic degradation [49]. The causes of this behavior are not clarified; however, the authors suggest that it must be attributed to more causalities than fast degradation and should be investigated.

Studies using linezolid as the delivery drug are numerous. Many alternatives have used this antibiotic in release to prevent infections related to orthopedic implants. Although a burst release has been found in some of them [50,51,52], some recent studies have achieved a sustained release [53,54,55].

### 2.4. Microbiological Assays

The selection of the right concentration of each antibiotic was analyzed in the following results of the biofilm inhibition response against *S. aureus* ATCC29213 of the formulations loaded with different concentrations.

**Figure 7:**
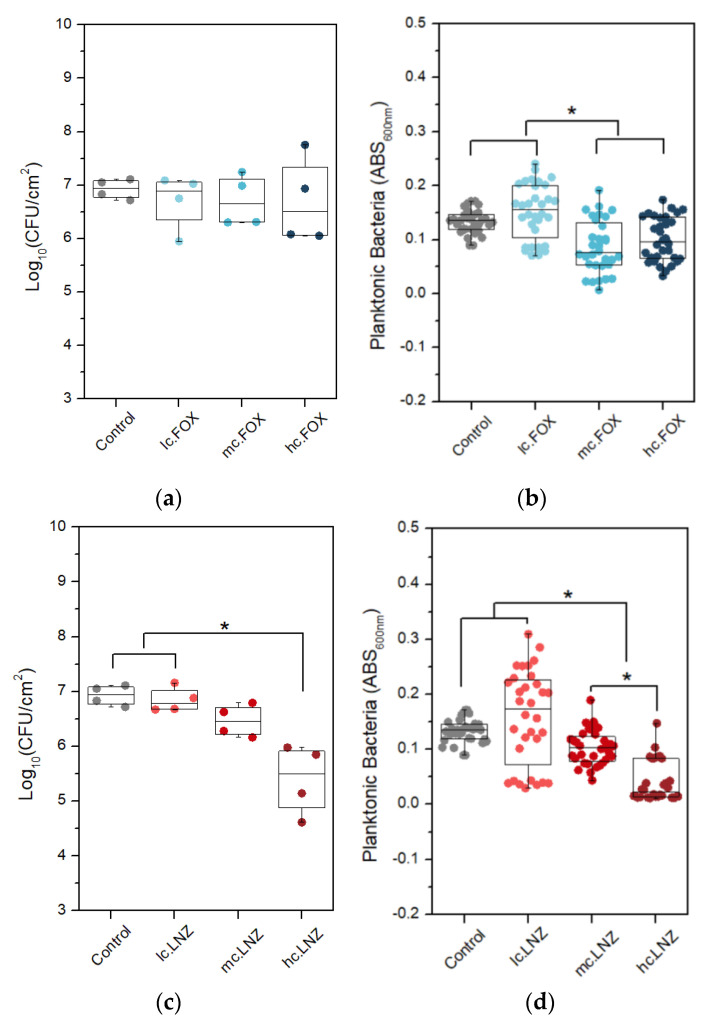
*S. aureus* colony-forming units per square centimeter (**a**,**c**) and planktonic bacteria (**b**,**d**) following strain incubation of titanium discs coated with Control, FOX-loaded coatings (**a**,**b**), or LNZ-loaded coatings (**c**,**d**). Data are represented as median and interquartile range. * *p*-value < 0.05 for Wilcoxon test.

Figure 7a,c show plots of the bacteria concentration per area unit that are attached to the surface as colony-forming units per square centimeter (CFU/cm^2^), while Figure 7b,d show the planktonic bacterial concentration, which is proportional to the absorbance of the supernatant measured at 600 nm. LNZ loading showed a concentration-dependent effect while FOX loading did not prevent biofilm formation. Similar behavior was observed when evaluating the reduction of planktonic bacteria in the *S. aureus* strain. In LNZ samples, the coating loaded with the maximum concentration (hc.LNZ) caused the greatest inhibition in biofilm development in *S. aureus*, inhibiting by up to 96% (Figure 7c). Moreover, hc.LNZ was effective at reducing the generation of planktonic bacteria by up to 83% (Figure 7d). In both cases, medium and high concentrations of antibiotics produced a reduction of planktonic bacteria with significant differences compared to the control. Comparing the two antibiotics, LNZ caused a higher reduction in planktonic bacteria than FOX.

While a slight inhibitory effect on the growth of planktonic bacteria was observed in coatings with higher concentrations of FOX, there was no inhibition of sessile bacteria in any of the FOX formulations. This could be caused by a combination of factors. On the one hand, part of the released antibiotic lost its bactericidal effect during sol–gel synthesis, possibly because it has been chemically bound to the sol–gel network. On the other hand, the effective antibiotic released does not reach inhibitory amounts in the initial hours. Being in the presence of sub-inhibitory concentrations of cefoxitin, biofilm formation is allowed. The effect found in planktonic bacteria compared to bacteria adhered to the surface can be explained by the fact that beta-lactams, such as FOX, are not active against biofilm [56], due to the acidic pH inside the biofilm, which these facultative anaerobic bacteria develop in their fermentative metabolism [57,58].

Based on these results, we decided to choose the highest concentrations of both antibiotics, because in the LNZ-loaded coatings, hc.LNZ showed greater biofilm inhibition, and in the FOX-loaded coatings, a better response of hc.FOX is expected when combined with LNZ.

The following results analyze the antibacterial effectiveness of the sol–gel coating loaded with both antibiotics (hc.FOX-LNZ), and for comparison, the Control, hc.FOX, and hc.LNZ coatings also participated in the assays. 

**Figure 8:**
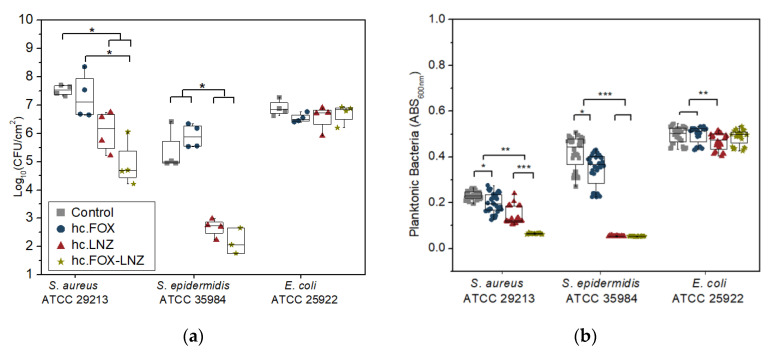
Quantification of (**a**) biofilms and (**b**) non-adherent planktonic bacteria of titanium pieces coated with Control, hc.FOX, hc.LNZ, and hc.FOX-LNZ. Data are represented as median and interquartile range of the log_10_(CFU/cm^2^) estimated by (**a**) drop plate and (**b**) absorbances (absorbance units, AU) obtained in four independent experiments. * *p*-value < 0.05, ** *p*-value < 0.01, *** *p*-value < 0.001 for Wilcoxon test.

An evaluation of the prevention of biofilm formation (Figure 8a), showed that hc.FOX-LNZ was effective in inhibiting biofilm formation in all strains except for *E. coli*. The greatest inhibition in biofilm development was shown against *Staphylococcus* species, obtaining 99.86% against *S. aureus* and 99.88% against *S. epidermidis* in the coating loaded with both antibiotics. The prevention of *E. coli* biofilm development was not efficient. 

When evaluating the reduction of the generation of non-adherent planktonic bacteria (Figure 8b), a behavior very similar to the inhibition of biofilm formation was observed. For *S. aureus* and *S. epidermidis* strains, hc.LNZ caused a higher reduction in planktonic bacteria than hc.FOX (48% against 15% for *S. aureus* and 88% against 17% for *S. epidermidis*). The planktonic bacteria reduction action of the coatings with both antibiotics showed a synergistic effect against *S. aureus*, but not against *S. epidermidis*. The linezolid-loaded coating caused a slight reduction in planktonic bacteria against *E. coli*. Although linezolid MICs for Gram-negative bacteria are higher than those for Gram-positive cocci, it is known that the ribosomes of *Escherichia coli* are as susceptible to linezolid as those of Gram-positive cocci [59].

The LNZ-loaded coatings inhibited the biofilm formation of *S. epidermidis* to a higher degree compared to *S. aureus*. This effect occurs because linezolid is more selective for *S. epidermidis* than *S. aureus.* This fact may be due to the difference in the generation time of each staphylococcus since; while the *S. epidermidis* generation time ranged between 17 and 38 min on surfaces [60], the *S. aureus* generation time is 20 min [61]. Although the combination of both antibiotics did achieve a greater decrease in biofilm formation for *S. aureus*, reaching a significantly greater reduction in planktonic bacteria compared to the formulations loaded with the antibiotics separately, the same effect was not achieved against *S. epidermidis*. What is more, FOX did not show any bactericidal effect against *E. coli*. Possible reasons for the inactivity of cefoxitin-loaded coatings were hypothesized in the above results. One possible explanation is that part of the concentration of FOX lost its antimicrobial effect by chemically bonding with some element of the sol–gel synthesis and the release of the rest of the antibiotic is of sub-inhibitory concentrations in the initial hours. The behavior against *E. coli* is the least favorable considering that the generation time of this strain is the shortest among those studied (only 20 min) [62]. The synergistic effect observed in the hc.FOX-LNZ coating against *S. aureus* demonstrates that not all FOX concentrations should have been affected.

In the studies found in the literature related to cefoxitin release, Kim et al. achieved inhibition of *S. aureus* growth of more than 90% with respect to the control [48]. The concentrations tested in this article were 0.04 mg/mL, 0.03 mg/mL, and 0.02 mg/mL. However, in the study by Back et al., no bacterial inhibition was shown at a 40% *w*/*w* concentration at any time point [49].

As a precedent of this work, this sol–gel system was used to design a moxifloxacin-loaded organic–inorganic sol–gel to prevent bacterial prosthetic joint infections. The microbiological study revealed that this coating completely inhibited the formation of biofilm development and treated a mature biofilm of the three evaluated bacterial species (*S. aureus, S. epidermidis, and E. coli*) [29].

The sol–gels described here will have the advantage of reducing the probable antibiotic resistance emergence related to antibiotic monotherapy.

The results obtained make this type of sol–gel coating a local treatment for OPRI in primary implantation surgeries or surgical approaches after bacterial infection where a one- or two-stage replacement is required. However, this approach would not replace the oral administration of antibiotics as systemic therapy but would rather reinforce it. Additionally, it could be considered to prevent infection of maxillofacial and dental implants.

### 2.5. Cellular Studies

**Figure 9:**
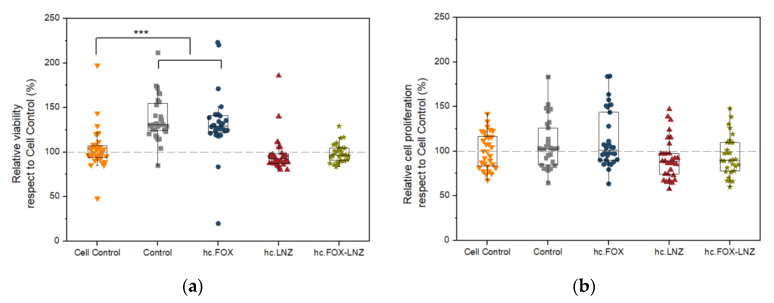
Percentage of MC3T3-E1 (**a**) viability and (**b**) proliferation without treatment (Cell Control) or treated with Control, hc.FOX, hc.LNZ, or hc.FOX-LNZ sol–gel coatings. Data are represented as median and interquartile range of the percentage of viability and cellular proliferation, respectively. in 32 biological replicates. *** *p*-value < 0.001 for Wilcoxon test.

Cell viability is not affected by the presence of any of the coatings studied (Figure 9a). In addition, there were no statistically significant differences in relative cell proliferation concerning the control (Figure 9b). These results are in line with previous works where they assert that this kind of non-antibiotic-loaded sol–gel can enhance the osseointegration of the implants coated with it [63], even when they are loaded with moxifloxacin [29]. This biocompatibility is desired in both orthopedic surgery [64] and dentistry [65,66].

### 2.6. Research Limitations

The findings of this study have to be viewed in light of some limitations. Firstly, the stability of antibiotics can condition the determination of their release kinetics. Linezolid undergoes a coloration change when in dissolution for prolonged periods at a physiological temperature [46,47]. Regardless of the coloration change, antibiotic hydrolysis degradation usually occurs under these circumstances [67]. Choosing absorbance as the method of measurement is perhaps not the most appropriate alternative. The release of the antibiotics in the coating loaded with both cannot be determined because the peaks of maximum absorbance for FOX and LNZ are very close. Likewise, the degradation of the sol–gel influences the determined absorbance values, and although the Control coating is considered to determine its contribution to the measurement, the degradation of the loaded and unloaded coatings are not the same. It is suggested to use high-performance liquid chromatography (HPLC) to avoid the above limitations; however, the degradation of antibiotics will occur regardless of the measurement method used. Secondly, the microbiological assays were performed with laboratory reference strains and were not extended to individual clinical isolates strains. Therefore, not only must the experimental conditions be considered but the behavior of these strains may also be different from those found in patients with OPRI. The reference strains present genomic differences concerning clinical strains and might have lost important pathophysiological characteristics since the bacteria adapt very quickly to in vitro conditions [68]. Thirdly, further studies are required to confirm the efficacy of these sol–gels using in vivo models.

## 3. Conclusions

The studies corresponding to coating degradation, antibiotic release, biofilm inhibition, cell viability, and proliferation were carried out satisfactorily in coatings loaded with cefoxitin and/or linezolid in different concentrations.

Even though in the first 24 h there are not many signs of degradation of the studied coatings, it is enough to obtain a constant release of both antibiotics. Linezolid release is sufficient to cause greater than 90% biofilm inhibition for *S. aureus* and *S. epidermidis*, but cefoxitin release is compromised. One plausible explanation is that part of the cefoxitin concentration lost its antimicrobial effect by chemically bonding during the sol–gel synthesis and the release of the rest of the antibiotic is of sub-inhibitory concentrations in the initial hours. The synergistic effect observed in the hc.FOX-LNZ coating against *S. aureus* demonstrates that not all cefoxitin concentrations were affected.

The coating loaded with a higher concentration of linezolid and the coating loaded with both antibiotics showed an excellent bactericidal and anti-biofilm response to staphylococcal species, without compromising cell viability and proliferation.

Although the introduction of cefoxitin has not been successful in preventing infections in Gram-negative species, at least in the strain tested, the use of the coating loaded with both antibiotics is interesting as it achieves greater staphylococcal inhibition, avoiding the resistance emergence in these species.

## 4. Materials and Methods

### 4.1. Sol–Gel Synthesis and Coatings Preparation

Hybrid organic–inorganic sol–gel coatings were synthesized according to a previously published methodology [42]. These were designed from the combination of the organopolysyloxanes methacryloxypropyltrimethoxy silane (MAPTMS, 98%, Acros Organics, Thermo Fisher Scientific, Waltham, MA, USA) and tetramethyl orthosilane (TMOS, 98%, Acros Organics, Thermo Fisher Scientific, Waltham, MA, USA) and were biofunctionalized with tris (tri-methylsilyl)phosphite (92%, Sigma–Aldrich, St. Louis, MO, USA). In the formulations, the molar ratio of MAPTMS to TMOS was fixed at 1:2, and silanes and the phosphorus precursor were in a 50:1 molar ratio. Ethanol and water were added to the reaction in stoichiometric amounts. Before addition to the component mix, the antibiotics were dissolved or suspended in water, although some of the ethanol used in the formulation contributed to the dissolution or improved solubility of the antibiotics. Three coatings were loaded with the following concentrations of linezolid (LNZ, Acros Organics, Thermo Fisher Scientific, Waltham, MA, USA): 0.61 (lc.LNZ), 1.23 (mc.LNZ), and 2.46 (hc.LNZ) mg/mL; alternatively, they were loaded with cefoxitin (FOX, Sigma-Aldrich, St. Louis, MO, USA) in the following concentration: 0.81 (lc.FOX), 1.62 (mc.FOX), and 3.24 (hc.FOX) mg/mL. Sol–gel functionalized with organophosphite without the addition of antibiotics was used as a control. Moreover, a formulation was produced and loaded with both antibiotics (hc.FOX-LNZ) in the following concentrations: 3.24 mg/mL of cefoxitin and 2.46 mg/mL of linezolid. All reagents were mixed before the addition of water. Once the aqueous solution had been added dropwise, suspensions were stirred for 24 h in a glove box. All reagents were used as received.

The sample preparation for the studies of coating degradation, antibiotic release rate, and microbiological assays was conducted as described below. The samples consisted of titanium substrates of 15 mm diameter × 2.5 mm thickness, prepared by a conventional powder metallurgy route, where the deposition of the sol–gel coatings was performed by dip coating at a rate of 200 mm/s. Afterward, samples were dried at 60 °C for 60 min inside an oven.

A series of analytical trials focused on studying the crosslinking and the surface of these coatings was developed in a previous report [42].

### 4.2. Study of Sol–Gel Degradation

The coating degradation of the evaluated systems was assessed on a Metrohm Autolab PGSTAT302N potentiostat (Metrohm; Herisau; Swiss) and a three-electrode configuration, using the coated TiPM substrate as the working electrode, a saturated silver/silver chloride reference electrode (E = 0.197 V vs. NHE), and a platinum wire counter electrode. A PBS solution (Sigma-Aldrich, St. Louis, MO, USA) at 37 ± 1 °C was used as the electrolyte. To guarantee the temperature, a double-walled glass cell was used to recirculate water at 37 °C through the cell wall using the Julabo HC/F30 temperature control system (Julabo UK Ltd., Stamford, UK). 

To carry out these measurements, the samples required prior preparation. TiPM disks were attached to a copper wire on the uncoated side using conductive tape. The measurement area (a coating measurement area of 1 cm^2^) was delimited by covering the rest of the disk with an edge retention lacquer (MacDermid, Waterbury, CT, USA). A frequency sweep was performed from 10 mHz to 10 kHz applying a sinusoidal signal of ±10 mV amplitude to the open circuit potential (OCP). For each formulation, at least 3 replicates were performed, and each sample was immersed in PBS at 37 °C, recording the EIS curves every 2 h for 24 h and measuring the OCP before and after each impedance. Interferences were avoided by using a Faraday cage. The impedance spectra were obtained and analyzed using Nova v2.1.4 and Z-view v3.3e software, respectively.

### 4.3. Kinetics Study of Antibiotics Release

Absorbance measurements for the kinetics study of antibiotics release were performed using a JASCO V-650 UV–vis absorption spectrophotometer (JASCO Corporation, Tokyo, Japan). Coatings were exposed to 3 mL of deionized water (DI, Sigma-Aldrich, St. Louis, MO, USA) at 37 °C and placed in 50-mL Falcon^TM^ conical tubes (Thermo Fisher Scientific, Waltham, MA, USA). Measurements were made in triplicate. The cefoxitin and linezolid release were monitored by measuring the maximum absorbance of both antibiotics (235 nm [69,70] and 251 nm [71,72,73], respectively) at different times (2, 4, 6, 12, 24, and 48 h). For each timepoint, 600 µL aliquots were extracted and transferred to a 700 µL quartz cuvette (10 mm pathlength, Hellma, Essex, Germany). Aliquots were replaced by new DI. Obtaining the concentration of the antibiotics was determined by employing the calibration curves and the absorbance values obtained. Calibration was previously performed for each antibiotic in DI, varying the concentration of both antibiotics between 35 × 10^−3^ µg to 35 µg. The concentration of each sample was then recalculated according to the following factors: The control coating contribution, the dilution performed at each timepoint, and the calibration curve. The calibration curves were linear over the measured concentration range with an R^2^ = 0.9998 for the cefoxitin calibration curve and R^2^ = 0.9967 for the linezolid calibration curve.

### 4.4. Evaluation of Biofilm Formation Inhibition

The first study was completed to choose the minimum concentrations of each antibiotic corresponding to the coatings with the greatest biofilm inhibition response against the methicillin-susceptible *S. aureus* from the American Type Culture Collection ATCC 29213 strain. The chosen concentrations were used to synthesize a sol–gel formulation loaded with both antibiotics simultaneously.

Subsequent studies analyzed the antimicrobial behavior of the coating without antibiotics (Control) and the coatings loaded with the chosen concentrations of each antibiotic separately, and in the same formulation. These experiments were performed using the reference strains *S. aureus* ATCC 29213, *S. epidermidis* ATCC 35984, and *E. coli* ATCC 25922. These species represent the most common pathogens related to prosthesis-related infections and constitute susceptible strains to these antibiotics.

Before the start of the experiments, all strains were preserved under freezing conditions at −80 °C. They were then maintained at 37 °C in tryptic soy agar + 5% sheep blood plates (TSS, BioMérieux, Marcy-l’Étoile, France). The day before each test, a TSS plate spread of the corresponding strain was made to be incubated overnight at 37 °C and 5% CO_2_. Each assay was performed in 4 independent experiments.

The biofilm formation inhibition assay was performed by immersing the coated TiPM discs in 5 mL of a solution diluted to a final concentration of 0.5 McFarland (≈1.6 × 10^8^ colony-forming units per milliliter, CFU/mL) of the chosen strain in the tryptic soy broth medium (TSB, Sigma Aldrich, St. Louis, MO, USA) supplemented with 1% glucose (*w*/*v*) in wells of a 6-well plate (Sigma Aldrich, St. Louis, MO, USA). They were left in immersion for 24 h at 37 °C and 5% CO_2_. After incubation, discs were rinsed with a 0.9% NaCl sterile saline solution (SS, B. Braun, Melsungen, Germany) and coatings were scraped off using sterile halved tongue depressors that were then sonicated at RT for 5 min [74] in 5 mL of SS. The drop plate method was then used to determine CFU/cm^2^ values [75]. Additionally, absorbance at 600 nm was used in eight repetitions to quantify the number of non-adherent planktonic strains still present in the incubation medium, using TSB media alone as a negative control. EnSpire Multimode Plate Reader (Perkin Elmer, Waltham, MA, USA) equipment was used for absorbance measurements.

### 4.5. Cell Study

In 96-well plates with α-minimum essential media containing 10% bovine fetal serum and 1% penicillin-streptomycin (αMEM, Invitrogen, Thermo Fisher Scientific, Waltham, MA, USA), MC3T3-E1 cells were seeded at a concentration of 10,000 cells/cm^2^ before being incubated at 37 °C and 5% CO_2_ overnight. After incubation and cell adhesion had occurred, the medium was replaced by αMEM with 50 mg/mL ascorbic acid (Sigma–Aldrich, St. Louis, MO, USA) and 10 mM ß-glycerol-2-phosphate (Sigma–Aldrich, St. Louis, MO, USA) for osteoblastic differentiation. With this medium, the plate lid was replaced with an MBEC^TM^ biofilm incubator lid (Innovotech, Edmonton, AB, Canada) previously prepared with the formulations. On the previous day, the MBEC^TM^ biofilm incubator lid pegs were dipped into wells containing 200 µL of each sol–gel formulation (negative control, Control, hc.FOX, hc.LNZ, and hc.FOX-LNZ, *n* = 32 for each) and allowed to dry in a laminar flow hood. Using this MBEC^TM^ biofilm incubator lid, they were incubated for 48 h at 37 °C in 5% CO_2_. After this time, cytotoxicity was determined using a CytoTox 96^®^ NonRadioactive Cytotoxicity Assay (Promega, Madison, WI, USA) and cell proliferation using the AlamarBlue^®^ solution (BIO-RAD, Hercules, CA, USA) at 10% (*v*/*v*) by measuring fluorescence intensity at excitation and emission wavelengths of 540 and 600 nm, respectively, in a Tecan Infinite 200 Reader (Tecan Group Ltd., Männedorf, Switzerland).

### 4.6. Statistical Analysis

Statistical analyses were performed using the non-parametric Kruskal–Wallis test or the one-sided pairwise Wilcoxon non-parametric test using IBM^®^ SPSS^®^ Statistics software release 26.0.0.0 (IBM Corp., Endicott, NY, USA), considering different significant levels of statistical significance. All trials involved at least three replicates. Data are represented in the graphs as the mean and standard deviation or as the median and interquartile range depending on the distribution of the data as normal or non-normal. 

## Figures and Tables

**Table 1:** Constant release rate up to 6 h and maximum concentration released reached for coatings.

	Constant Release Rate up to 6 h (µg/h)	Concentration Released (µg)
lc.FOX	0.75	8.96
mc.FOX	1.47	21.51
hc.FOX	3.21	31.36
lc.LNZ	0.75	5.78
mc.LNZ	1.67	16.73
hc.LNZ	3.71	33.47

## Data Availability

Not applicable.
